# The impact of peer support work on the mental health of peer support specialists

**DOI:** 10.1186/s13033-022-00561-8

**Published:** 2022-10-18

**Authors:** Daniel Poremski, Jonathan Han Loong Kuek, Qi Yuan, Ziqiang Li, Kah Lai Yow, Pui Wai Eu, Hong Choon Chua

**Affiliations:** 1 Health Intelligence Unit, Institute of Mental Health, Buangkok Green Medical Park, 10 Buangkok View, Singapore, 539747 Singapore; 2 Research Department, Institute of Mental Health, Singapore, Singapore; 3 Department of Nursing, Institute of Mental Health, Singapore, Singapore; 4 Department of Allied Health Services, Institute of Mental Health, Singapore, Singapore; 5 Department of General Psychiatry, Institute of Mental Health, Singapore, Singapore; 6 Office of the CEO, Institute of Mental Health, Singapore, Singapore

**Keywords:** Peer support services, Health care quality, Qualitative methods

## Abstract

**Background:**

Peers support specialists have positive impacts on the mental health of their service users. However, less is known about how their mental health changes as a result of their activities.

**Methods:**

We followed 10 peer support specialists over their first year of employment and interviewed them thrice. We used grounded theory to analyse the way in which the health of participants changed.

**Results:**

Self-reported mental health of our participants did not change over the course of the study. However, the role did help participants grow and learn about their condition and their strengths. While sharing their past experiences could be taxing, they learned how to harness their recovery journey without risking relapse.

**Conclusion:**

Entering the role of a peer support specialist does not appear to negatively impact mental health, but might enhance insight and resilience. However, this appears to occur in individuals who already possess an inclination toward introspection.

**Supplementary Information:**

The online version contains supplementary material available at 10.1186/s13033-022-00561-8.

## Introduction

Mental health services are increasingly integrating peer support workers into their practices. Their wider integration is recommended on the grounds of their contribution to the recovery of service users experiencing various psychiatric illnesses [1–7]. The addition of peer support workers improves the outcomes of certain interventions, such as in the treatment of addictions [8], in assertive community outreach teams [9–12], in supported employment settings [13]. However, their use in general psychiatry as agents delivering interventions is less well-defined and less supported by evidence [5, 14]. Reviews have concluded that peer support services produce results similar to services-as-usual control conditions [5, 15–18]. Qualitative research echoes these conclusions. Paulson et al. [19] found that peers provide a similar structure of services compared to non-consumers, with the exception of a greater focus on empowerment and recovery. This is to be expected as in certain situation peers may model their actions on the way they have seen others act when integrated into new teams [20].

Integration of peer support workers into clinical settings has not been without its challenges. Barriers continue to limit the impact of such services. Numerous factors severely impede a peer’s ability to effectively carry out their role, including: lack of clarity of the peer’s role within broader mental health services, discrimination and prejudice from non-peer co-workers, lack of relevant additional training opportunities, low pay, inadequate basic training, and the constant need to maintain their wellbeing [21–25]. These barriers lead to higher rates of discontinuation amongst peer workers, alienation by non-peer staff, and burnout [21, 25].

While most research on peer workers has focused on the implementation, integration, and development of peer workers [26–29], relatively few have sought to understand how introducing peer workers into a new working environment impacts the peer support worker and their mental health. The literature that does exist observes that the wellbeing of peers may be influenced by the relationships they develop, by the challenges they encounter while managing boundaries, and by the act of reliving personal storied while supporting others [23]. Along with this knowledge comes concern for the health of people working as peers, especially from colleagues [30]. Hence, our aim was to determine if and how the peer support worker role impacts the mental health of peer support workers.

## Methods

This study was part of a larger mixed-methods study designed to assess the global impact of peer support services [31]. To determine if the mental health of our Peer Support Specialists (PSS) changed over time, we chose to rely on self-reported experiences. Qualitative data provides more information about the nature of the changes compared to quantitative data, such as service use data. We followed the Consolidated criteria for reporting qualitative research (COREQ), an atheoretical framework, to guide the reporting of our study and its findings [32]. Ethics approval was granted by institutional (# 646-2018) and national ethic review committees (#2018/01131). All participants gave written informed consent and were given the option at each interview to decline audio recording.

The theoretical framework underpinning the larger project was derived from previous work focusing on service fidelity [33], which sought to assess the “active ingredients” of PSS services. Much of the interview questions were based on the content of Chinman’s preliminary fidelity scales in order to ensure that the interview covered activities identified as central to peer services. As a result of this alignment with the fidelity scale, we asked participants to speak about their experiences of modelling recovery, sharing their story, engaging service users, promoting hope, teaching coping skills, and working in multidisciplinary teams. This framework also allowed us to discuss whether any particular “active ingredient” influenced mental health.

### Setting

The Institute of Mental Health is the largest and predominant source of psychiatric care in Singapore. Numerous PSS are active throughout the institute in various departments. Recently, PSS have been introduced to an acute female ward, with unfruitful attempts to introduce another to a male acute ward. The duties they complete at the institute vary by department but generally include using lived experience with services to help existing service users. They are integrated into the clinical teams and participate in case discussions and ward rounds. A loose job description at the institutional level was formulated to allow departments to tailor the duties of the peers to their department’s needs. This loose description also allowed their job scope to evolve [31]. Disclosure of lived experiences is left to the discretion of the PSS.

### Sampling

We received ethics approval to speak with all staff employed at our institute under the title of Peer Support Specialist. Because of the limited pool of people from which to draw participants, we elected to allow recruitment of people who had been employed as PSS with the institute for up to 2 years. Prior to that time, the institute had yet to adopt a proactive policy of developing and integrating PSS into the workforce, and the experiences of PSS hired prior to this watershed might be different. Additional recruitment dispositions can be found in a previous project manuscript [31] and in the Additional file 1.

## Interview process

We conducted the interviews between April 2019 and April 2020. We scheduled three individual interviews separated by 4-month gaps. The interview guide was based on the fidelity scale and the accompanying body of literature that served as the theoretical foundation of the project. It contained questions designed to echo the open-ended format captured by the elements of the fidelity scale. It also included questions germane to the assessment of the PSS services and their mental health. The three interviews contained similar questions, but the first focused more on their recovery journey, and the latter two focused on the things that had changed. All the interviewers were conducted by the same researcher to leverage previously established rapport. The interviewer reflected upon interviews and significant topics after each interview and producing memos linked to interviews. On average, interviews lasted 63 min (SD 20 min).

## Reflexivity

It would be false to report that the authorship team was disinterested in the success of PSS services, as each author has an interest in the advancement of the PSS movement in Singapore, and the recovery movement in general [22, 34]. While this is the case, the study’s present purpose of determining the impact of PSS work on those performing it can be achieved in a way that is not negatively affected by the team’s interest. We are also cognizant that any bias that leads to ignoring challenges faced by our participants would be deleterious to the health of the PSS and to the overall movement. The first author, who conducted the interviews, does not have a managerial role in the oversight of PSS. As such, we believe the participants were not obliged to give answers that may advance or hinder those involved in the project or the PSS program. We believe that the content of the interviews was sincere. Finally, none of the authors would benefit or suffer from the success or failure of the program.

## Data analysis

Recorded interviews were transcribed verbatim and coded by the first and second authors using NVivo 11 [35], a software that allows researcher to track, organize, and analyse the content of interviews. With this tool, content can be coded and linked to themes of interests to show relationships and similarities. Coding was done inductively by the first and second authors, and progressed as the interviews were conducted. This allowed for an iterative process to occur where new codes could be incorporated into subsequent interviews, along a constant comparative method. Furthermore, the longitudinal design of our study allowed for these preliminary codes and themes to be shared and discussed with the participants, which provided greater confidence in their veracity, as proposed by Grossoehme & Lipstein [36]. The present article relies heavily on sections of the interviews where the conversation turned specifically to the way adopting the PSS role impacted participants. We used a grounded theory approach [37] to explain how assuming the role of a PSS impacted their health. Content was given importance based on its frequency and its intensity. For example, many participants spoke of the importance of reflexivity (frequency). But fewer mentioned the dangers of generalising the impact of PSS on improved health, but they spoke about it at length (intensity). Verbatim quotes, followed by a participant identifier, are provided to support the proposed theory.

## Results

Our 10 participants ranged in age from 22 to 41 at the time of their employment as PSS, with a mean of 30 years. Six were women and four were men. Their diagnoses varied and matched the department in which they were placed, for example those in the mood disorders departments had mood disorders. Three had schizophrenia-related diagnoses, three had major depression, two had bipolar disorder, one had an addiction-related diagnosis, and the last had an obsessive–compulsive disorder. No participant dropped out of the study.

## Did PSS impact mental health?

Without exception, participants spoke very favourably of their health as it related to the PSS work demands and stressors. They all saw ways in which the role helped them grow.

PSS made me be more aware of taking care of my own health. I learn the importance of self-care after knowing that I need to be healthy in order to help others. Overall it has generated a positive impact on my life. 50008.

While there were several instances when participants described stressors and setbacks that they attributed to the obligations of employment (working with co-workers and delivering services) rather than to specific PSS obligations, these stressors they believed could be reasonably seen as universal amongst office workers.

… I think everyone goes through stress and I think it’s about knowing what works for you and when challenges come and knowing how to manage. 50009.

Or be related to the nature of the job

… serious down sides? Yes a bit, because I get triggered a bit more when I am inside the ward, sometimes I get thoughts about certain things that have passed already, like suicide, so a lot of things give me a bit more unwell sometimes, but I think it is the nature of the job, it is triggering but I can cope with it slowly 50010.

These views also allowed them to feel a sense of normalcy and pride in being able to overcome their challenges despite having to endure symptoms associated with a mental health condition. It allowed them to rewrite and reframe their narratives from a more positive perspective.

However, most participants then indicated that they also knew of people who had not been well.

Because again, it’s a business, it’s a service that we’re providing, and because we’re based on this principle of like recovery and everything. And I think definitely we’ve seen PSSs who are not doing as well but they will not [seek services], they were afraid of the kind of like the repercussions of saying “I’m not well”. 50005.

While we were unable to access people with this experience, the fact that the participants saw and knew of such bad experiences was an important point which they felt obliged to share.

## How PSS led to better health?

The way performing PSS duties contributed to the health of the PSS varied, but could be linked back to a few skills related to reflexivity. Participants who reflected on their illness were able to grow by reflecting on their experiences:

- Participant: I think [my health] is more positive. I really take care of my own thoughts, my own process of things, even people negative with you, sarcastic with you, very negative with you, you learn how to deal with it.
- Interviewer: how do you learn? Like where does that learning come from?
- Participant: through the course of helping others, for me I will have to think where that motivation, where that source of energy come from. And if is come from some spirituality or some internal voice, I think that part really make a lot of sense, yeah. And in order to help others, you yourself have to be well. I can’t be doing unethical thing, secretive thing, and yet putting another front in front of others. […] Previously my thought wasn’t that. My thought was like covering up, putting the best foot forward to show others how well you are, but inside is all injured. 50003.

But being a PSS did not invariably give them that ability, it existed in them prior to assuming their PSS role. This is captured in the quote below stressing the importance of having essential skills prior to commencing PSS work.

That is when I always say that PSS staff should ALWAYS learn what they are doing. It is a job, it is a skilled job, it is not a job where you come in and then learn how to be a PSS. No, you learn how to be a PSS first, and then you come into the job, it is a skill, […] we cannot do that because we cannot make mistakes while on the job because it involves human beings and their emotions. 50007.

Self-care, closely followed the idea of reflective thinking.

but it made me realise the importance of self-care. […] There are very different things and you realise how important it is to have efficient self-care so you can be there for someone else. 50,009.

 Yeah, because otherwise what’s going to happen is even if you let’s say fake that you’re okay, it down the line, you’re going to show signs that you’re not doing okay. You’re going to show signs that you’re unhappy, you’re breaking down, you’re not socialising people as much, you withdraw from crowds, and that kind of thing. So self- care is very important in this role. And you have to be very mindful of that. 50002.

Participants warned that expecting this introspective skill to emerge in an individual who lacks it might put the person at risk, and in their mind explained why some people find the job challenging.

If they are neglecting self-care in any matter, in any course, I think then they would not make a good PSS. […] Nothing stigmatising about it, but if they do not know how to take care of themselves, then how are they going to support their fellow peers? 50007.

The job may even deceive certain PSS, making it harder for them to take care of themselves in the event of a relapse.

However, some became more in touch with their emotions and perspectives because of their roles as PSS. Even though the work was challenging, it provided plenty of growth opportunities and allowed for PSS to become “stronger” and more “confident”.

I think it has been good. Because from the start actually, I learned quite a lot, so I have grown quite a lot since day one, a lot emotionally physically and in health. I think I have learned to be stronger in handling certain issues and I am quite confident in handling issues now, but at the same time it is quite draining for me at certain times, especially mid-year. 50010.

PSS interviewed shared that part of being a role-model was to present an authentic portrayal of themselves and their conditions. This required that they be honest with themselves about their health. They did not feel that this pressure to be a role model interfered with their help seeking, but rather that it led them to embrace the fact they sought assistance as a sign of self-awareness. They could perform their function despite not being well all the time.

I think being a role model is not pretending I’m totally well, it’s to be truthful to my peers, so all my peers know that if I’m going down, going up, going down, I’ll tell them, even my inpatient, outpatient, I will tell them. 50006.

Developing this self-honesty was one way in which adopting the PSS role helped people learn when seeking services was imperative and when they could rely on their own resilience to weather the storm.

## Avoiding generalisations

While it surfaced that being employed as a PSS had several beneficial effects, some considered the implications and the risks. Participants considered the risks of encouraging people with lived experience to pursue a PSS career as a means of helping them recover, and discouraged the generalisation of the conclusion.

“I know for some people it helps them, but I don’t think that we should generalise it. Because for a lot of us, we didn’t choose to become a PSS for that reason. 50004”

Furthermore, given the nature of the dual role PSS hold, as a service user and provider, some PSS identified how being a role model for others could lead to detrimental outcomes for their health. This consequence was due to the PSS’s desire and sense of responsibility to be a helping professional who did not need to seek professional services on the side.

We will always feel that as a PSS I need to be very strong, I need to be well, I need to make everything contain and doing so good in my wellness. I see that as another detrimental way that we may not want to reveal our condition, how are we really coping with the fear of losing the job or not being a good role model to our peers. 50001.

This sense of professionalism could hinder their ability to seek help whilst in their role of a PSS as it constantly caused dissonance between their desire to remain well and continuing advocating for others to seek help, while not doing the very thing they were espousing not to appear weak.

So eventually I did go and when I was waiting for the medication at the pharmacy, I actually teared, but it’s not teared of feeling weak about myself, but I just feel so, it’s like being an advocate myself, like telling the caregivers “please ask your loved ones to seek help” and now me myself doing it is so tough, what much more about their loved ones. 50001.

The idea that PSS work was not suitable for anyone with lived experience is somewhat against the opinion of some advocates that share the belief that anyone can be a PSS. However, one PSS reconciled these seemingly opposing views by noting that the type of support or aid someone can provide may differ depending on their stage of recovery:

I think there are many things you can still do, even if it is not through individual sessions, there is always a way to contribute, whether it is group work, I don’t know, or even sharing your experiences, yeah coming up with programmes, there are so many ways that you could do it, and I think sometimes we think PSS should look a certain way, but it is also about exploring how it should look, especially where we are and who we work with, in this context. 50009.

This essentially reminds us that peer support exists on a continuum, from being structured certified service providers embedded into clinical teams to being informal mutual support group members. Each manifestation of peer-support has its challenges and contributions.

## Discussion

Our goal was to explore if and how the role of a PSS impacted the mental health of those in the role. A natural extension of talking about the impact and the benefits of this specialised work was the warning of generalising conclusions. PSS work did lead to benefits and personal growth, but pre-existing reflexive capabilities supported this growth. We learned that in general, being a PSS did not jeopardize their health. Adopting the role and being employed allowed them to experience a sense of normality and benefitted their health via the psychological satisfaction of inclusivity of employment and team membership. Acting as a peer to those with acute illness helped them better understand their own condition.

Being able to experience life stressors and manage them effectively allowed PSS to feel a sense of belonging to and as a part of a more extensive care system. Reflexivity stood out as being a facilitator of this process. Many PSS in our study believed that the ability to reflect upon their experiences of supporting their clients was essential. Our finding echoes those identified in studies seeking to identify strategies to improve the implementation of peer support programmes in the mental health sector [38]. Perhaps, introducing mentorship schemes could also serve to support this reflexivity as more senior PSS could serve as clinical supervisors for new PSS [26]. This may accelerate the development of reflexive skills.

Supportive colleagues and nurturing supervisors were also essential to the PSS’s health. Our participants felt they could share their issues openly, and felt supported during times of personal crisis. Working with people who genuinely cared about them as both individuals and colleagues helped PSS feel a sense of warmth and belonging. This strong support emerged as an element of successfully implemented peer support services, according to large surveys of American services [20]. Being in an Asian community, where family ties and connections are treasured [39, 40], these close relationships serve as a buffer from daily stressors and allow for greater psychological wellbeing. Conversely, alienation has been a consistent barrier to the implementation of peer-supported services [21]. The benefits our participants derived from strong professional connections further supports the notion that more should be done to integrate PSS into teams [41].

Our findings indicate that acting as a role model, a considerable part of the PSS raison-d’etre [42], served as a double-edged sword. While some PSS in our study saw it as an unavoidable burden, some held opposing views and saw its potential to generate greater authenticity and forge more genuine connections with their clients. As the nature of their job pulls heavily from their lived experience, added training to better cope with the boundaries and unpack issues related to the concept of role-modelling could be beneficial. This knowledge would allow PSS to harness their greatest asset better and mitigate the potential for it to become a negative influence on their health.

A word of caution is warranted. While our participants derived benefits from their activities, suggesting that people with lived experience should more generally pursue PSS roles may be unadvisable. The stressors of adopting the PSS role may exceed the coping skills of some people in recovery, despite their wish to adopt the PSS role. This is in contrast to the opinion of some peer support advocates that anyone can be a PSS. To reconcile these two opinions, we must be mindful that there are various types of peer support that exist, from mutual support groups to certified service providers. It is likely that people at various stages of recovery may be able to provide a suitable and beneficial level of contribution to others regardless of whether they are certified and imbedded in clinical teams, such as our service providers. Consequently, organisations will likely benefit from expanding various types of peer support suitable for people at various stages of recovery, to allow those who wish to contribute, but who do not yet meet the criteria for PSS certification, to none-the-less share their experiences in a structured and safe mutually-supportive environment.

### Limitations

It may be possible to view our decision to adopt a self-report approach to health as fallible. Relying on structured clinical interviews to determine if the health of our participants changed over time might have led to different conclusions. However, the process of accepting our participants’ own assessment of their health is in line with the recovery movement and self-determinism.

Our sampling framework does lead to potential limitations. We spoke exclusively with people who successfully graduated from the certification program, had successfully obtained employment in a large mental health institute, and had demonstrated a level of proficiency. It was not feasible to approach all people who enrolled in the certification programme, or in other words, all the people with ambition to become a PSS. Our initial objective appeared very relevant from the institute’s perspective: to ensure that the health of its employees was maintained. However, our ultimate conclusion should be viewed with the following distinction in mind: There is a possible difference between the impact of working as a PSS, compared with the impact of attempting to become a PSS. Based on our participants’ experiences, the latter perspective may be more relevant to the community, whereas the former might be too focused on the health of employees. Hence, future studies are required to determine if our findings are isolated to large mental health institutes or whether they generalize to the community and to other settings.

## Conclusion

Peer work, although novel in most Asian contexts, has the potential to fill a gap in existing mental health services. Furthermore, the health benefits are not limited to the people receiving peer services but extend to the peer workers as well. Adopting the role of a PSS may help those who already possess some self-reflexive tendencies to extend the skill and improve their self-awareness and understanding. However, we must be cautious not to assume everyone will flourish in a PSS role, and must continue to improve our understanding of the various factors that influence the mental health of PSS while they endeavour to support others.

## Supplementary Information

**Additional file 1.** Additional description of the Setting, Sampling and Interview process.

## Data Availability

Raw data is not available because of the confidentiality of the interviews and regulations dictating the conditions under which the data could be ethically collected. The qualitative data contains personal details which may lead to the identification of participants. Participants did not explicitly consent to the sharing of their data with third parties.

## References

[1] Barr KR, Townsend ML, Grenyer BFS (2020). Using peer workers with lived experience to support the treatment of borderline personality disorder: a qualitative study of consumer, carer and clinician perspectives. Borderline Personal Disord Emot Dysregul.

[2] Gordon J, Bradstreet S (2015). So if we like the idea of peer workers, why aren't we seeing more?. World J Psychiatry.

[3] Muralidharan A, Peeples AD, Hack SM, Fortuna KL, Klingaman EA, Stahl NF, Goldberg RW (2020). Peer and non-peer co-facilitation of a health and wellness intervention for adults with serious mental illness. Psychiatr Q.

[4] Nestor P, Galletly C (2008). The employment of consumers in mental health services: politically correct tokenism or genuinely useful?. Australas Psychiatry.

[5] Repper J, Carter T (2011). A review of the literature on peer support in mental health services. J Ment Health.

[6] Schlichthorst M, Ozols I, Reifels L, Morgan A (2020). Lived experience peer support programs for suicide prevention: a systematic scoping review. Int J Ment Health Syst.

[7] Wusinich C, Lindy DC, Russell D, Pessin N, Friesen P (2020). Experiences of parachute NYC: an integration of open dialogue and intentional peer support. Community Ment Health J.

[8] Humphreys K, Wing S, McCarty D, Chappel J, Gallant L, Haberle B, Weiss R (2004). Self-help organizations for alcohol and drug problems: toward evidence-based practice and policy. J Subst Abuse Treat.

[9] Bond GR, Drake RE, Mueser KT, Latimer E (2001). Assertive community treatment for people with severe mental illness: critical ingredients and impact on patients. Disabil Manag Health Outcomes.

[10] Sells D, Davidson L, Jewell C, Falzer P, Rowe M (2006). The treatment relationship in peer-based and regular case management for clients with severe mental illness. Psychiatr Serv.

[11] White H, Wehelan C, Barnes JD, Baskervill B (2003). Survey of consumer and non-consumer mental health service providers on assertive community treatment teams in Ontario. Community Ment Health J.

[12] Wright-Berryman JL, McGuire AB, Salyers MP (2011). A review of consumer-provided services on assertive community treatment and intensive case management teams: implications for future research and practice. J Am Psychiatr Nurses Assoc.

[13] Kern RS, Zarate R, Glynn SM, Turner LR, Smith KM, Mitchell SS, Liberman RP (2013). A demonstration project involving peers as providers of evidence-based, supported employment services. Psychiatr Rehabil J.

[14] Mahlke CI, Kramer UM, Becker T, Bock T (2014). Peer support in mental health services. Curr Opin Psychiatry.

[15] Davidson L, Bellamy C, Guy K, Miller R (2012). Peer support among persons with severe mental illnesses: a review of evidence and experience. World Psychiatry.

[16] Davidson L, Chinman M, Lkloos B, Weingarten R, Stayne D, Tebes JK (1999). Peer support among individuals with severe mental illness: a review of the evidence. Clin Psychol Sci Pract.

[17] Davidson L, Chinman M, Sells D, Rowe M (2006). Peer support among adults with serious mental illness: a report from the field. Schizophr Bull.

[18] Simpson EL, House AO (2002). Involving users in the delivery and evaluation of mental health services: systematic review. BMJ.

[19] Paulson RI, Post RL, Herinckx HA, Risser P (2002). Beyond components: using fidelity scales to measure and assure choice in program implementation and quality assurance. Community Ment Health J.

[20] Foglesong D, Spagnolo AB, Cronise R, Forbes J, Swarbrick P, Edwards JP, Pratt C (2022). Perceptions of supervisors of peer support workers (PSW) in behavioral health: results from a national survey. Community Ment Health J.

[21] Adams WE (2020). Unintended consequences of institutionalizing peer support work in mental healthcare. Soc Sci Med.

[22] Kuek JHL, Chua HC, Poremski D (2021). Barriers and facilitators of peer support work in a large psychiatric hospital: a thematic analysis. Gen Psychiatr.

[23] Mirbahaeddin E, Chreim SA (2022). Narrative review of factors influencing peer support role implementation in mental health systems: implications for research, policy and practice. Adm Policy Ment Health.

[24] Otte I, Werning A, Nossek A, Vollmann J, Juckel G, Gather J (2020). Challenges faced by peer support workers during the integration into hospital-based mental health-care teams: Results from a qualitative interview study. Int J Soc Psychiatry.

[25] Shalaby RAH, Agyapong VIO (2020). Peer support in mental health: literature review. JMIR Ment Health.

[26] Jenkins GT, Shafer MS, Janich N (2020). Critical issues in leadership development for peer support specialists. Community Ment Health J.

[27] Jones N, Kosyluk K, Gius B, Wolf J, Rosen C (2020). Investigating the mobility of the peer specialist workforce in the United States: Findings from a national survey. Psychiatr Rehabil J.

[28] Kent M (2019). Developing a strategy to embed peer support into mental health systems. Adm Policy Ment Health.

[29] Klee A, Chinman M, Kearney L (2019). Peer specialist services: New frontiers and new roles. Psychol Serv.

[30] Roennfeldt H, Byrne L (2020). How much ‘lived experience’is enough? Understanding mental health lived experience work from a management perspective. Aust Health Rev.

[31] Poremski D, Kuek J, Qi Y, Li Z, Yow KL, Eu PW, Chua HC (2021). A longitudinal qualitative analysis of the way peer support specialist roles change over time in a psychiatric hospital setting in Asia. Adm Policy Ment Health.

[32] Tong A, Sainsbury P, Craig J (2007). Consolidated criteria for reporting qualitative research (COREQ): a 32-item checklist for interviews and focus groups. Int J Qual Health Care.

[33] Chinman M, McCarthy S, Mitchell-Miland C, Daniels K, Youk A, Edelen M (2016). Early stages of development of a peer specialist fidelity measure. Psychiatr Rehabil J.

[34] Kuek JHL, Liang AG, Goh TW, Poremski D, Su A, Chua HC (2021). The personal recovery movement in Singapore—past present, and future. Ann Acad Med Singap.

[35] NVivo 11 Plus (64-bit). QSR International Pty Ltd. 2017. http://www.qsrinternational.com/.

[36] Grossoehme D, Lipstein E (2016). Analyzing longitudinal qualitative data: the application of trajectory and recurrent cross-sectional approaches. BMC Res Notes.

[37] Glaser BG, Strauss AL (2009). The discovery of grounded theory: strategies for qualitative research.

[38] Baxter L, Fancourt D (2020). What are the barriers to, and enablers of, working with people with lived experience of mental illness amongst community and voluntary sector organisations? A qualitative study. PLoS One.

[39] Kuek JHL, Raeburn T, Wand T (2020). Asian perspectives on personal recovery in mental health: a scoping review. J Ment Health.

[40] Pathare S, Kalha J, Krishnamoorthy S (2018). Peer support for mental illness in India: an underutilised resource. Epidemiol Psychiatr Sci.

[41] Byrne L, Roennfeldt H, Wolf J (2022). Effective peer employment within multidisciplinary organizations: model for best practice. Adm Policy Ment Health.

[42] Watson E (2019). The mechanisms underpinning peer support: a literature review. J Ment Health.

