# Supplementary material for: The impact of peer support work on the mental health of peer support specialists
Source: Int J Ment Health Syst. 2022 Oct 18;16:51. doi: 10.1186/s13033-022-00561-8 (PMC9578199; doi:10.1186/s13033-022-00561-8)
Supplement: Supplementary file 1 — Additional file 1. Additional description of the Setting, Sampling and Interview process. [file 13033_2022_561_MOESM1_ESM.doc]

# Setting

The institute of mental health was one of the first institutes in Singapore to employ peer support specialists (PSS). Amongst the first departments to employ PSS were the sections dedicated to the treatment of addiction, first episode psychosis, and mood disorders. The institute also contains departments dedicated to delivering psychotherapy, psychosocial rehabilitation, case management, community-based services, occupational therapy, outpatient and emergency services, all of which employ one or more PSS. Recently, PSS have been introduced to an acute female ward, with unfruitful attempts to introduce another to a male acute ward. In 2016, the first cohort of 23 people with lived experience began the PSS certification programme jointly developed by the host institute the National Council for Social Services and the Singapore Association of Mental Health. Several of the programme graduates joined the host institute in 2017, marking an important local benchmark in the development of peer support services. The programme has continued to certify PSS.

# Sampling

Concerning the justification for excluding PSS who were hired prior to the specified watershed: People who were employed by the institute before 2017 had different experiences as their placement, hiring and selection were determined more by the needs of individual departments. Prior to 2017, the institute had yet to adopt a proactive policy of developing and integrating PSS into the workforce, explaining the selection criteria of the study.

We did not seek to recruit or interview people who had unsuccessfully completed the PSS certification program, or who had chosen to work in a PSS role outside the institute. The former exclusion centred on our intention of determining the impact of PSS work on those who performed the job instead of determining the impact of completing PSS certification. The latter exclusion centred on the specific type of work done in our institute, where PSS are integrated into clinical teams, as compared to PSS working outside the institute, where they usually are not integrated into clinical teams.

### Interview process

Each PSS was followed for eight months and was interviewed at three points (baseline, four months and eight months). The interview guide was based on the fidelity scale and the accompanying body of literature that served as the theoretical foundation of the project. It contained questions designed to echo the open-ended format captured by the elements of the fidelity scale. It also included questions germane to the assessment of the PSS services and their mental health. The three interviews contained similar questions, but the first focused more on their recovery journey, and the latter two focused on the things that had changed over the period that had lapsed since the previous interview. All the interviewers were conducted by the same researcher to leverage previously established rapport. Notes were taken during the interviews and were reflected upon afterwards by producing memos linked to interviews. Participants spoke for an average of 67.8% of the interview (SD 15.2), and the interviewer spoke the rest of the time at 32.2% (SD 9.3).
